# The use of mass cytometry (CyTOF) to evaluate the cellular uptake of stable radiopharmaceutical surrogates in single cells: a proof-of-concept study

**DOI:** 10.1186/s41181-026-00442-2

**Published:** 2026-04-01

**Authors:** Miguel Gómez-Sánchez, Elisa Blanco-González, María Montes-Bayón, Martin Behe, Roger Schibli, Elisa Rioja-Blanco

**Affiliations:** 1 Department of Physical and Analytical Chemistry, Faculty of Chemistry, University of Oviedo, Julián Clavería 8, 33006 Oviedo, Spain; 2 Instituto de Investigación Sanitaria del Principado de Asturias (ISPA), Av. Hospital Universitario s/n, 33011 Oviedo, Spain; 3 Center for Radiopharmaceutical Sciences, PSI Center for Life Sciences, Villigen-PSI, Switzerland; 4 Department of Chemistry and Applied Biosciences, Institute of Pharmaceutical Sciences, ETH Zurich, Zurich, Switzerland

**Keywords:** Mass cytometry, CyTOF, Radiopharmaceuticals, Targeted radionuclide therapy

## Abstract

**Background:**

Current methods for assessing radiopharmaceutical uptake are based on scintillation counting (e.g., γ-counting) or imaging techniques, such as positron emission tomography (PET) and single photon emission computed tomography (SPECT), which evaluate the average radiopharmaceutical uptake in biological samples. Single-cell analysis of radiopharmaceutical uptake and biodistribution would provide a better understanding of the possible heterogeneous uptake in cellular populations and organs, with the potential to improve current dosimetry and toxicity assessments. In this proof-of-concept study, we evaluate the use of mass cytometry (CyTOF) as an analytical tool to study the uptake of non-radioactive radiopharmaceutical surrogates at the single-cell level.

**Results:**

The preclinical immunoconjugate DOTA-cAC10, targeting CD30 (a receptor overexpressed in lymphomas), was labeled either with radioactive lutetium-177 (^177^Lu) resulting in the [^177^Lu]Lu-DOTA-cAC10 radioimmunoconjugate, or stable lutetium-175 (^175^Lu), yielding the surrogate [^175^Lu]Lu-DOTA-cAC10. For CyTOF experiments, cells were incubated with the stable surrogate and an iridium DNA intercalator (Cell-ID™) to enable concurrent determination of immunoconjugate uptake and cell identification. CyTOF analysis of the surrogate was performed in three T-cell lymphoma cell lines with varying CD30 expression (Karpas 299, Myla and Jurkat) and compared to γ-counting data obtained for the radioimmunoconjugate. Radioimmunoconjugate [^177^Lu]Lu-DOTA-cAC10 cellular uptake studies by γ-counting showed receptor-dependent accumulation, with Karpas 299 cells exhibiting the highest uptake levels (32.5 ± 1.6% of total added activity), followed by Myla (20.0 ± 1.0%) and Jurkat cells (15.7 ± 1.2%). Mass cytometry enabled analysis of the stable surrogate [^175^Lu]Lu-DOTA-cAC10 uptake at single-cell resolution, revealing median signal intensities of ^175^Lu of 32.2, 14.0, and 2.94 a.u. for Karpas 299, Myla, and Jurkat cells, respectively.

**Conclusions:**

The sensitivity of CyTOF enabled discrimination of uptake between cell models even at low metal-to-antibody stoichiometric ratios. It also revealed intercellular variability in uptake that can only be captured with single-cell methods. Overall, we showed that CyTOF is a robust, high-throughput, multiplexed approach for characterizing the cellular uptake of stable radiopharmaceutical surrogates at single-cell resolution, paving the way for future studies.

**Supplementary Information:**

The online version contains supplementary material available at 10.1186/s41181-026-00442-2.

## Background

The use of radiopharmaceuticals in targeted radionuclide therapy (TRT) is a rapidly evolving area of research with the potential to revolutionize cancer treatment (Varghese et al. 2024, Sgouros et al. 2020, Lepareur et al. 2023). Various targeting moieties can be used for this purpose, including small molecules, peptides, and antibodies. In the last decade, the peptide-based radiopharmaceuticals Pluvicto^®^ ([^177^Lu]Lu-PSMA-617) and Lutathera^®^ ([^177^Lu]Lu-DOTATATE), both employing the β¯-emitter lutetium-177 (^177^Lu), have been approved for the treatment of metastatic castration-resistant prostate cancer and gastroenteropancreatic neuroendocrine tumors, respectively (Hennrich et al. 2019, Sallam et al. 2024). In this context, characterization of radiopharmaceutical cellular uptake and biodistribution is critical for evaluating its pharmacodynamics, optimizing dosimetry, and improving overall therapeutic efficacy. Conventional methods for assessing these parameters include scintillation counting (e.g., γ-counting) and non-invasive imaging techniques (positron emission tomography, PET, and single photon emission computed tomography, SPECT), which lack single-cell resolution (Nelson et al. 2024, Lawhn-Heath et al. 2022). These techniques measure the average radionuclide accumulation on a population of cells, limiting the ability to detect differences in radiopharmaceutical uptake among individual cell types within heterogeneous populations. This is particularly relevant in tumors, which consist not only of malignant cells but also of diverse immune, stromal, and endothelial cell populations (Bejarano et al. 2021). Bulk analysis methods fail to capture cell-level heterogeneity in radiopharmaceutical distribution, limiting the accurate evaluation of accumulation in non-tumor tissues.

Recently, alternative methods to conventional radioactive approaches have been explored in radiopharmacy, mainly through the use of inductively coupled plasma mass spectrometry (ICP-MS) (Klika et al. 2024). ICP-MS has gained attention as a highly sensitive, multi-element and multi-isotope technique for the determination of compounds in biological samples, either via their natural containing elements, or via artificial labeled elemental tags (Klika et al. 2024, Liu et al. 2017). Therefore, this technique represents an interesting alternative for evaluating radiopharmaceutical uptake and biodistribution by using stable isotope-labeled radiopharmaceutical analogs (Wallimann et al. 2023, Wallimann et al. 2024). As it eliminates radiation exposure and reduces radioactive waste, it is well suited for laboratories without radiation protection infrastructure. Although ICP-MS has recently been applied to characterize radiopharmaceuticals using stable isotope-labeled surrogates, its use has so far been limited to bulk analysis, providing only average elemental content across cell populations rather than single-cell resolution (Klika et al. 2024, Wallimann et al. 2023, Wallimann et al. 2024). Even though single-cell analysis by elemental mass spectrometry (SC-ICP-MS) has been applied for several years in various biological contexts, such as the quantification of Pt-based chemotherapeutic uptake in tumor cells (Corte-Rodríguez et al. 2020, Corte et al. 2017), it has not yet been employed for radiopharmaceutical characterization.

Mass cytometry, also known as cytometry by time-of-flight (CyTOF), combines the single-cell analysis principles of fluorescence-based flow cytometry with the selectivity and quantitative power of ICP-MS (Ornatsky et al. 2010, Bendall et al. 2012). This powerful combination allows simultaneous detection of up to 50 parameters within individual cells, making it an invaluable tool for immunophenotyping, signaling pathway analysis, and cell state characterization in fields such as immunology, oncology, and drug discovery (Tanner et al. 2013, Atkuri et al. 2015). In CyTOF, the fluorophores used to label antibodies in conventional flow cytometry are replaced by stable metal isotopes, commonly lanthanides, due to their natural absence in biological systems (Majonis et al. 2010). This enables the detection of a higher number of parameters than conventional flow cytometry avoiding the spectral overlap, and providing greater resolution and sensitivity (Bendall et al. 2012, Iyer et al. 2022). The signal obtained for each isotope corresponds to a specific labeled-antibody and consequently correlates with the antigen levels within individual cells. To maximize detection sensitivity, Maxpar^®^ technology enables labeling of antibodies with up to 100 metal isotopes, allowing detection of even low-abundance antigens per cell. Prior to the analysis, cells are fixed, permeabilized, and stained with a DNA intercalator (e.g., iridium-193, ^193^Ir) to identify nucleated singlets and discriminate them from debris and non-single-cell events, including doublets and cell aggregates. The cell suspension is nebulized into droplets containing single cells, which are vaporized, atomized, and ionized in an argon plasma. The resulting ion clouds are filtered by a quadrupole to remove low-mass ions (< 75 Da) and analyzed by time-of-flight (TOF) mass spectrometry, where ion counts are detected and converted into electrical signals (Ornatsky et al. 2010, Bendall et al. 2012, Iyer et al. 2022).

Mass cytometry is widely used for cellular immunophenotyping in both clinical and basic research, however its potential for evaluating the single-cell distribution of radiopharmaceuticals has not yet been explored (Iyer et al. 2022, Chuah and Chew 2020, Zhang et al. 2020, Maby et al. 2020). In this proof-of-concept study, we investigated the use of mass cytometry to assess radiopharmaceutical cellular uptake with single-cell resolution. As a model radiopharmaceutical, we employed the immunoconjugate DOTA-cAC10, consisting of an antibody targeting CD30 (a validated therapeutic target for lymphoma), conjugated to the chelator DOTA enabling the radiolabeling with ^177^Lu. The radioimmunoconjugate [^177^Lu]Lu-DOTA-cAC10 has shown promising preclinical results in lymphoma mouse models (Rioja-Blanco et al. 2025). Cellular uptake was evaluated by γ-counting using the ^177^Lu-labeled radioimmunoconjugate and by mass cytometry using its stable surrogate [^175^Lu]Lu-DOTA-cAC10. Since radioimmunoconjugates must avoid excessive antibody modification to prevent impaired antigen binding and unfavorable biodistributions (Delage et al. 2021, Knogler et al. 2006), and each DOTA chelator coordinates only a single lanthanide ion, the metal loading per antibody is limited, which may challenge the sensitivity of the mass cytometry assay. To investigate whether single-cell detection was feasible under these conditions, the cellular uptake of the stable surrogate was evaluated in three cell lines with varying CD30 expression levels. To our knowledge, this study represents the first application of mass cytometry to assess radiopharmaceutical uptake at single-cell resolution.

## Methods

### Antibody conjugation and metal labeling

The anti-CD30 chimeric monoclonal antibody cAC10 was produced by Proteogenix (Schiltigheim, France). Conjugation with the macrocyclic metal chelator DOTA was performed via random lysine coupling with the bifunctional chelating agent p-SCN-Bn-DOTA (Macrocyclics, USA, ID number B-205), based on the protocol developed by Cooper et al. (Cooper et al. 2006). In brief, a 1:15 antibody-to-chelator molar ratio was reacted in 0.1 M boric acid buffer, pH 9.0, for 15 h at room temperature. Excess of the chelating agent was removed by ultrafiltration (Vivaspin 6, Sartorius, Germany), and the immunoconjugate was buffer-exchanged into PBS, pH 7.4. The average number of DOTA molecules per antibody was six, as determined by liquid chromatography mass spectrometry (LC-MS) (Fig. S1).

For radiolabeling, no-carrier-added [^177^Lu]LuCl_3_ in 0.04 M HCl was obtained from ITM Medical Isotopes (Munich, Germany). Radiolabeling was performed by incubating 0.4 MBq of [^177^Lu]LuCl_3_ per microgram of the immunoconjugate DOTA-cAC10 in 0.25 M ammonium acetate buffer, pH 5.5, for 1 h at 37 °C, 350 rpm. EDTA (1 mM final concentration) was added and incubated for an additional 5 min to chelate residual free lutetium. The radioimmunoconjugate, [^177^Lu]Lu-DOTA-cAC10, was purified using PD-10 desalting columns (Cytiva, USA) and buffer-exchanged into PBS, pH 7.4. Specific activity and radiochemical purity were assessed by size exclusion chromatography (SEC) coupled to a radiodetector (Hitachi LaChrom Elite, Hitachi, Japan) using a Superose^®^ 6 10/300 GL column (Cytiva) for separation. PBS buffer, pH 7.4 was used as the mobile phase at an isocratic flow rate of 0.75 mL/min.

For labeling with stable ^175^Lu, a 50 µM [^175^Lu]LuCl_3_, 0.04 M HCl aqueous solution was freshly prepared and mixed with 100 µg DOTA-cAC10 in a ratio of 1:2 DOTA-to-metal in 0.25 M ammonium acetate buffer, pH 5.5. The mixture was incubated for 1 h at 37 °C, 350 rpm. After the incubation, the reaction was quenched by adding EDTA to a final concentration of 1 mM and incubated for an additional 5 min to chelate the remaining free lutetium. The concentration of the resulting stable surrogate, [^175^Lu]Lu-DOTA-cAC10, was determined by UV-Vis spectrophotometry using a NanoDrop™ 2000 spectrophotometer (Thermo Fisher Scientific, Bremen, Germany) and resuspended in PBS to a final concentration of 0.5 mg/mL. The [^175^Lu]Lu-DOTA-cAC10 surrogate was analyzed by SEC coupled to ICP-MS detection (SEC-ICP-MS) to confirm the successful labeling using an HPLC system (1260 Infinity II, Agilent Technologies, CA, USA) connecting on-line to a triple quadrupole ICP-MS instrument (iCAP MTX ICP-MS, Thermo Fisher Scientific). In each analysis an aliquot of 50 µL of the PBS sample solution was injected in a Superdex^®^ 200 Increase 10/300 GL column (GE Healthcare, IL, USA). Separation was carried out in isocratic mode using ammonium acetate solution (50 mM, pH 7.0) as mobile phase at a flow rate of 0.7 mL/min. Detection was performed by ICP-MS monitoring the signal of ^175^Lu^+^ in single quadrupole (SQ)-mode.

## Cell lines and cell culture

The T-cell lymphoma cell lines Jurkat, Myla and Karpas 299 were kindly provided by Dr. Christoph Schlapbach (Inselspital, Bern, Switzerland). Cells were cultured in Roswell Park Memorial Institute 1640 medium (RPMI 1640, Invitrogen, Fisher Scientific, Madrid, Spain) supplemented with 10% (v/v) fetal bovine serum (Gibco, Life Technologies, Madrid, Spain) and 1% (v/v) Penicillin-Streptomycin (Gibco, Life Technologies). Cultures were maintained at 37 °C in a humidified incubator at 5% CO_2_.

## Flow cytometry

CD30 cell expression in the T-cell lymphoma cell lines (Jurkat, Myla and Karpas 299) was evaluated by flow cytometry (Attune™ CytPix™ Flow Cytometer, Thermo Fisher Scientific) using an allophycocyanin (APC)-conjugated anti-human CD30 monoclonal antibody for fluorescent staining (clone BY88, BioLegend, London, UK) and the corresponding isotype control (clone MOPC-21, BioLegend). Data were analyzed using FlowJo software v10.9.0 (BD Life Sciences, OR, USA).

## Cellular uptake by γ-counting

To assess radioimmunoconjugate cellular uptake, 1 million Jurkat, Myla, or Karpas 299 cells were incubated with 8 ng/mL of [^177^Lu]Lu-DOTA-cAC10, either alone or in the presence of 10 µg/mL of unlabeled cAC10 for receptor blocking. Following a 30-minute incubation at room temperature, cells were collected and washed with PBS to remove the unbound radioimmunoconjugate (supernatant fraction). Subsequently, cells were washed with a glycine buffer (pH 2.2) (membrane-bound fraction) and lysed with NaOH, 1 M (internalized fraction). The radioactivity in each of the fractions was then measured using a γ-counter (Packard Cobra II, Model 5003). The activities of the membrane-bound and internalized fractions were summed to determine the total cellular uptake of [^177^Lu]Lu-DOTA-cAC10. The uptake results are expressed as a percentage of the total added activity.

## Cell preparation for single-cell analyses

Cell preparation for single-cell analyses was performed following the Maxpar^®^ Cell Surface Staining with Fresh Fix protocol for CyTOF XT™ (Standard BioTools, San Francisco, CA, USA). In this work, the protocol was modified by substituting Maxpar^®^ metal-conjugated antibodies with the stable surrogate, [^175^Lu]Lu-DOTA-cAC10. The optimal surrogate concentration was determined using the binding equation derived from the Langmuir isotherm:

1 $$ B = \frac{{B{\mathrm{max}} \cdot \left[ L \right]}}{{KD + \left[ L \right]}} $$

where *B* is the amount of ligand bound at equilibrium, *B*_max_ is the maximum number of binding sites, [*L*] is the free ligand concentration, and *K*_D_ is the equilibrium dissociation constant. A concentration of 4 µg/mL was established and validated in titration experiments using Karpas 299 cells. 1–3 million cells of each cell line were resuspended in Maxpar^®^ Cell Staining Buffer (CSB, Standard BioTools) and then were incubated with 4 µg/mL of [^175^Lu]Lu-DOTA-cAC10 at room temperature for 30 min. Following the incubation, cells were washed three times with 1 mL of CSB and then the fixation of the cells was carried out by adding 1 mL of 1.6% formaldehyde solution to each sample, followed by a 10-minute incubation at room temperature. Cells were then centrifuged, the supernatant was removed, and the pellet was homogenized in the residual volume. For DNA staining, required for event identification and singlet gating, cells were incubated with Cell-ID™ Intercalator-Ir (125 nM, Standard BioTools), prepared in Maxpar^®^ Fix and Perm Buffer (Standard BioTools). This buffer is optimized for cell fixation and permeabilization, which is necessary for Ir to penetrate the cells and intercalate the DNA. Samples were incubated overnight at 2–8 °C. Following incubation, cells were washed twice with CSB and twice with Maxpar^®^ Cell Acquisition Solution (CAS, Standard BioTools). Finally, cells were resuspended in CAS at a final concentration of 0.5–1 × 10^6^ cells/mL before analysis by mass cytometry. The suspensions obtained from Karpas 299 cells were additionally subjected to single-cell analysis using quadrupole-based ICP-MS.

## Single-cell analysis by mass cytometry

All mass cytometry measurements were performed on a CyTOF XT instrument (Standard BioTools). The instrument is equipped with a pneumatic nebulizer connected to a heated spray chamber, operating at 200 °C for complete aerosol droplet desolvation prior to introduction into the plasma. The sample introduction system consists of an autosampler using a two-valve combination that handles the sample introduction to the nebulizer and the washing of all tubing and nebulizer after each sample. Therefore, the sample introduction and cleaning between different sample is fully automatized. The samples are placed in vials in a refrigerated carrousel at 4 °C.

Before analysis, the instrument was calibrated using EQ Six Element Calibration Beads (Standard BioTools), polystyrene beads containing defined concentrations of six metal isotopes. Calibration was performed according to the manufacturer’s instructions to adjust critical parameters such as plasma torch X-Y position, voltages, and nebulization flow, ensuring optimal resolution, sensitivity, and transport efficiency for subsequent measurements. Immediately before analysis, cell samples stained as described previously were diluted to 0.5–1 × 10^6^ cells/mL in CAS containing 10% of EQ Six Element Calibration Beads (Standard BioTools) to enable inter-sample normalization and to serve as an internal quality control. The suspension was then loaded into a 300 µL loop and pumped into the nebulizer at a constant flow rate of 30 µL/min. This cell concentration allows to achieve an acquisition rate of approximately 500 cell events per second. Cell event detection is performed by the integrated CyTOF software. Following event detection, the ion counts for every event are summed, and the integrated intensities are compiled into a Flow Cytometry Standard (FCS) 3.0 file that is further processed using FlowJo software v10.8.1 (BD Life Sciences).

## Single-cell analysis by quadrupole-based ICP-MS

Single-cell quadrupole-based ICP-MS measurements were performed using the triple quadrupole instrument iCAP MTX ICP-MS (Thermo Fisher Scientific) working in the single quadrupole (SQ)-mode for ^175^Lu^+^ monitoring. The instrument was fitted with a High Efficiency Sample Introduction System (Glass Expansion, Weilburg, Germany) that consists of a High Efficiency MicroMist™ Nebulizer with an inner capillary tube of 110 μm and a high efficiency spray chamber with a microjet gas adapter that was designed to improve transport efficiency of cells using a sheath gas flow. Cells suspensions obtained from Karpas 299 cells after incubation with [^175^Lu]Lu-DOTA-cAC10 following the procedure described previously were diluted to a final concentration of 0.5–1 × 10^6^ cells/mL in water (18.2 MΩ cm) purified in a PURELAB flex 3 system (ELGA VEOLIA, Lone End, United Kingdom). Immediately after dilution cells were pumped into the ICP-MS at a flow rate of 10 µL/min using the syringe pump Fusion 100X (Chemix Inc., Stafford, Texas, USA) fitted with a 1 mL Hamilton syringe (Hamilton, Reno, NV, USA). The data were recorded in a time-resolved analysis mode during 2 min per analysis at a dwell time of 100 µs. These data were processed to extract cell events (short, transient signal pulses produced by a cell entering the plasma) from the background using an established iterative procedure (Iyer et al. 2025), based on averaging the entire data set and collecting all data points that are three standard deviations (3σ) above the mean. This process is repeated until no new events are found. Data processing was performed using the SPCal software v1.4.5.

### Statistical analysis

Statistical analysis was performed using GraphPad Prism 8 (version 8.3.1). The γ-counting data are represented as mean ± SD. A p-value ≤ 0.05 was considered statistically significant. Comparisons between groups were analyzed by one-way ANOVA with Tukey’s multiple comparison test. The degree of concordance between the results obtained by γ-counting and flow cytometry, and between mass cytometry and flow cytometry, was determined using Pearson’s correlation.

## Results

### Labeling of DOTA-cAC10

Radiolabeling of the immunoconjugate DOTA-cAC10 with ^177^Lu was achieved with high specific activities (≥ 0.3 MBq/µg). The resulting radioimmunoconjugate [^177^Lu]Lu-DOTA-cAC10 yielded radiochemical purities greater than 90%, as confirmed by SEC coupled to a radiodetector (Fig. S2).

For single cell quadrupole-based ICP-MS and mass cytometry experiments, DOTA-cAC10 was labeled with the stable isotope ^175^Lu and further analyzed by SEC-ICP-MS. The chromatogram (Fig. S3) showed a major peak at ~ 16 min corresponding to the [^175^Lu]Lu-DOTA-cAC10 stable surrogate, along with two minor ^175^Lu-containing species, representing immunoconjugate aggregates (~ 13 min) and another corresponding to ^175^Lu- EDTA (~ 28 min). EDTA was used to eliminate excess of uncomplexed ^175^Lu^3+^. Based on the column calibration and compound-independent detection by ICP-MS, approximately 70% of the eluted ^175^Lu was associated with the [^175^Lu]Lu-DOTA-cAC10 immunoconjugate (Fig. S3).

### Cellular uptake by γ-counting

**Fig. 1:**
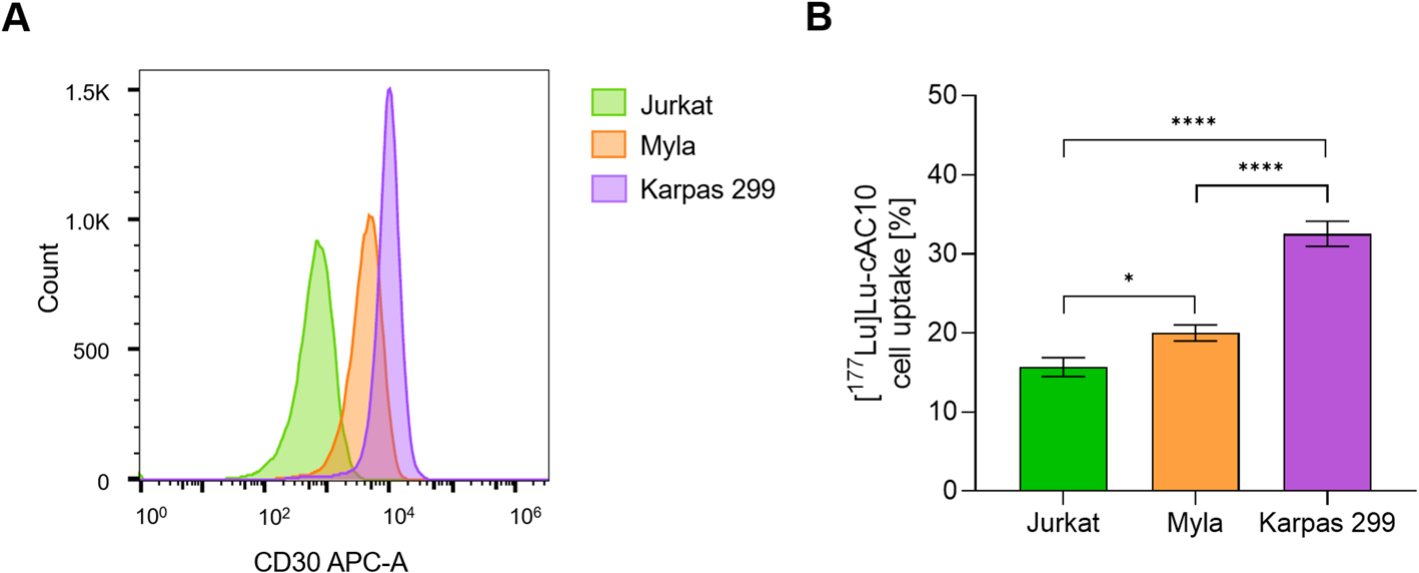
CD30 expression and [^177^Lu]Lu-DOTA-cAC10 uptake in T-cell lymphoma cell lines. A) Flow cytometry overlay histograms for CD30 expression on the indicated T-cell lymphoma cell lines (Jurkat, in green; Myla, in orange; Karpas 299, in violet). B) [^177^Lu]Lu-DOTA-cAC10 cell uptake in the tested cell lines by γ-counting (mean ± SD,* n=3*). * *p ≤ 0.05*, **** *p ≤ 0.0001* (one-way ANOVA, Tukey’s multiple comparison test).

Cellular uptake of the radioimmunoconjugate [^177^Lu]Lu-DOTA-cAC10 was evaluated in three cell lines showing different levels of CD30 expression, as determined by flow cytometry (Fig. 1A, Fig. S4). Jurkat cells showed the lowest CD30 expression with a median fluorescence intensity (MFI) value of 756, Myla cells exhibited an intermediate expression (MFI of 4626), and Karpas 299 cells displayed the highest levels of the target CD30 receptor (MFI of 10048).

Cellular uptake of [^177^Lu]Lu-DOTA-cAC10 after 30 min of incubation was measured by γ-counting (Fig. 1B, Fig. S5). The lowest uptake was observed in Jurkat cells (15.7 ± 1.2% of the added activity), followed by Myla cells (20.0 ± 1.0% of the added activity), while Karpas 299 cells showed the highest radioimmunoconjugate accumulation (32.5 ± 1.6% of the added activity). These uptake levels correlated with the CD30 membrane expression in these cell lines, yielding a Pearson correlation coefficient (r) of 0.984.

### Cellular uptake at the single-cell level by mass cytometry

In order to study the cellular uptake of the stable surrogate [^175^Lu]Lu-DOTA-cAC10 at the single-cell level, the standard Fluidigm Maxpar^®^ Cell Surface Staining with Fresh Fix protocol for CyTOF XT was adapted by substituting Maxpar^®^ metal-conjugated antibodies with [^175^Lu]Lu-DOTA-cAC10. To select an appropriate concentration of [^175^Lu]Lu-DOTA-cAC10 for these experiments, the aim was to remain right at the saturation concentration to be able to assess differences in uptake among the three cell lines under study. The *K*_*D*_ of the radioimmunoconjugate [^177^Lu]Lu-DOTA-cAC10 in Karpas 299 cells was previously determined as 3.5 nM (95% CI: 2.9–4.1 nM) (Rioja-Blanco et al. 2025). Based on this data, the saturation concentration, defined as 90% CD30 receptor occupancy, was calculated to be 31.5 nM (95% CI: 26.1–36.9 nM). Thus, an incubation concentration of approximately 25 nM (corresponding to 4 µg/mL) was selected to approximate a near-saturating concentration for CyTOF analysis. Under the experimental uptake conditions used in this study, ligand binding is not at equilibrium, thus these occupancy values should be considered theoretical estimates used to guide experimental conditions.

**Fig. 2:**
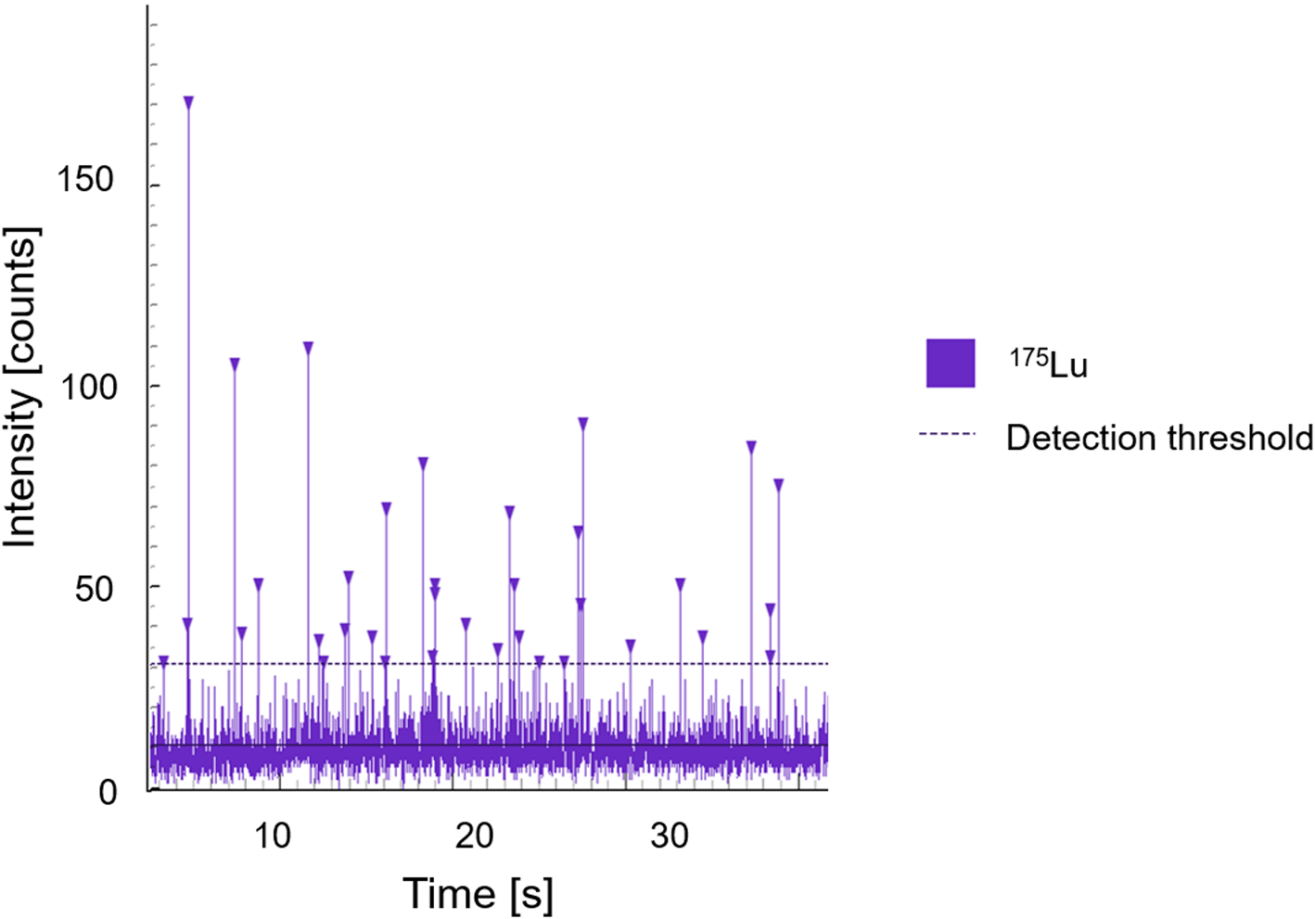
Single cell [^175^Lu]Lu-DOTA-cAC10 uptake in Karpas 299 cells. Single cell events registered for ^175^Lu^+^ by time-resolved single cell quadrupole-based ICP-MS analysis in Karpas 299 cells after incubation with the stable surrogate [^175^Lu]Lu-DOTA-cAC10.

Preliminary experiments were carried out using single-cell quadrupole-based ICP-MS in Karpas 299 cells to test the sensitivity of detection for [^175^Lu]Lu-DOTA-cAC10 at the selected concentration (Fig. 2). The intensity of the recorded events, each corresponding to a cell event by monitoring ^175^Lu^+^, was high enough to be distinguishable from the background noise. This intensity was greater than the established threshold for cell events determined by a previously reported iterative procedure (Corte et al. 2017). Therefore, by using a concentration of 4 µg/mL for [^175^Lu]Lu-DOTA-cAC10, the single-cell ICP-MS methodology demonstrated sufficient sensitivity to detect the lutetium signals corresponding to individual cell events for experiments with this stable radiopharmaceutical surrogate.

To define the experimental conditions for the subsequent CyTOF analysis, Karpas 299 cells were first incubated with both [^175^Lu]Lu-DOTA-cAC10 and the ^193^Ir-based DNA intercalator to conduct the simultaneous detection of the two isotopes within each cell event. As previously mentioned, the ^193^Ir-based DNA intercalator is used to identify single cell events and discriminate doublets, thus ensuring that the lutetium signals are assigned to single cells. To validate the concentration of [^175^Lu]Lu-DOTA-cAC10 selected, a titration experiment was first performed in Karpas 299 cells using increasing concentrations of [^175^Lu]Lu-DOTA-cAC10 (1–6 µg/mL). A concentration-dependent increase in the signal intensity of ^175^Lu in the Karpas 299 cells was observed up to 4 µg/mL. Further increases in concentration did not provide any increase in signal intensity and thus, the staining was conducted using 4 µg/mL (Fig. S6).

**Fig. 3:**
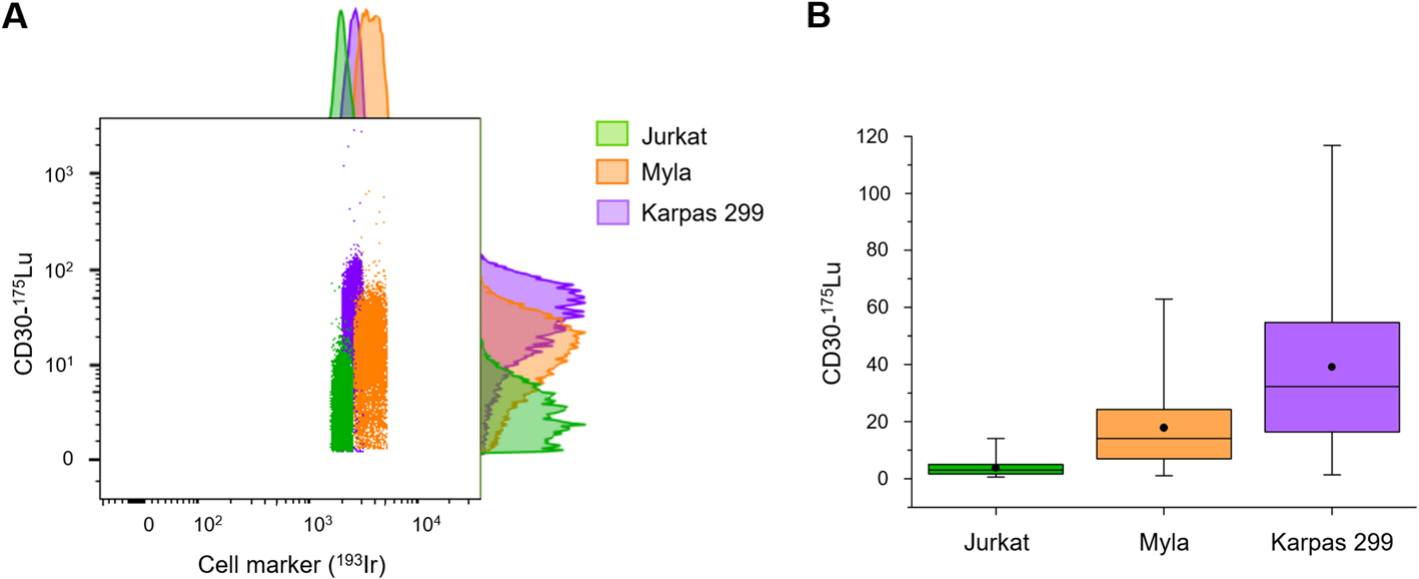
Dual monitoring of ^193^Ir and ^175^Lu in T-cell lymphoma cells lines using CyTOF. Jurkat (green), Myla (orange), and Karpas 299 (violet) cells were analyzed by CyTOF and results were represented as (A) two-dimensional dot-plots of ^175^Lu-CD30 vs. ^193^Ir-DNA (cell marker) and histograms, and (B) boxplots showing median (line), mean (dot), interquartile range (box width), and whiskers (range excluding outliers) of the intensities of the ^175^Lu signals in the analyzed cells (>5000 cells in all cases) for the three cell lines.

Following the establishment of staining conditions, the three T-cell lymphoma cell lines were incubated with [^175^Lu]Lu-DOTA-cAC10 (4 µg/mL) and the ^193^Ir-based DNA intercalator, followed by CyTOF analysis. The aim was to determine whether differences in radiopharmaceutical surrogate uptake among the different cell lines under study with different levels of expression of CD30 could also be detected at the single-cell level. CyTOF analysis confirmed that all cells were positively stained with the ^193^Ir-based DNA intercalator, while ^175^Lu signal intensities varied across cell lines, reflecting differences in the radiopharmaceutical surrogate uptake (Fig. 3A). When visualized as a boxplot, the distribution of ^175^Lu signal intensities showed clear differences among the three cell models (Fig. 3B). Karpas 299 cells exhibited the highest uptake, with a median ^175^Lu signal intensity of 32.2 a.u., followed by Myla cells (14.0 a.u.) and Jurkat cells (2.94 a.u.). These results strongly correlated (*r* = 0.999) with their respective levels of CD30 expression measured by fluorescent flow cytometry and were consistent with the bulk cellular uptake data obtained by γ-counting (Table 1). The stronger correlation between mass cytometry and flow cytometry data, compared with that between γ-counting and flow cytometry (*r* = 0.984), can be attributed to the differences in antibody concentration used in each method. For γ-counting experiments, a sub-saturating concentration of radioimmunoconjugate was used (8 ng/mL), whereas the stable surrogate in CyTOF experiments was applied at near-saturating concentration to ensure sufficient sensitivity (4 µg/mL), similar to flow cytometry where antibodies are added in excess. Under sub-saturating conditions, uptake remains preferential in cells with higher receptor expression, as demonstrated, but it is not linearly proportional to receptor per cell number. This explains the stronger correlation between CyTOF and flow cytometry absolute values (*r* = 0.999) compared to the ones obtained for γ-counting experiments. In addition, both cytometric techniques directly measure the number of cells binding the anti-CD30 antibody (labeled with ^175^Lu or with a fluorophore, respectively), whereas γ-counting infers the average cellular uptake as a fraction of the total activity added to the cells, further contributing to the higher discrepancies in the absolute values obtained for both methods. In any case, the correlation between mass cytometry and γ-counting is preserved, as higher receptor-expressing cells consistently show higher radioimmunoconjugate and surrogate uptake, supporting the suitability of mass cytometry for identifying preferential radiopharmaceutical accumulation across cell populations with different levels of target expression. Importantly, mass cytometry enabled the detection of stable surrogate uptake at the single-cell level, identifying subsets of cells with substantially lower or higher signal intensities than the median, which highlights an intercellular variability that is only detectable using single-cell techniques (Fig. 3).

**Table 1.** Summary of the CD30 expression and cellular uptake of [^175^Lu]Lu-DOTA-cAC10 and [^177^Lu]Lu-DOTA-cAC10. The table summarizes CD30 expression assessed by flow cytometry (single-cell analysis using fluorescent labels), and cellular uptake of [^175^Lu]Lu-DOTA-cAC10 and [^177^Lu]Lu-DOTA-cAC10 via mass cytometry (single-cell analysis) and γ-counting (bulk analysis), respectively.

Cell line	CD30 expression (median fluorescence intensity)	[^175^Lu]Lu-DOTA-cAC10 cell uptake (median ^175^Lu intensity)	[^177^Lu]Lu-DOTA-cAC10 cell uptake (percentage of added activity, mean ± SD) (%)
Jurkat	756	2.9	15.7 ± 1.2
Myla	4626	14.0	20.0 ± 1.0
Karpas 299	10,048	32.2	32.5 ± 1.6

## Discussion

The primary objective of this study was to evaluate the feasibility of mass cytometry as a novel tool to test radiopharmaceutical uptake at the single-cell level using non-radioactive radiopharmaceutical surrogates. Specifically, we aimed to determine whether differences in radiopharmaceutical surrogate cellular uptake could be detected at the single-cell level across cell lines with varying levels of target receptor expression. As a model, we used the preclinical radioimmunoconjugate [^177^Lu]Lu-DOTA-cAC10 targeting CD30, a receptor overexpressed in lymphomas. Its stable surrogate [^175^Lu]Lu-DOTA-cAC10 was used to study single cell uptake in three T-cell lymphoma cell lines with different expression of CD30 by mass cytometry. Given that stable isotopes of lanthanides are commonly used as metal tags for CyTOF applications and their radioactive counterparts serve as therapeutic radionuclides in TRT, mass cytometry represents an attractive technique for the characterization of radiopharmaceuticals (Han et al. 2018).

In conventional CyTOF workflows, antibodies are typically labeled using the Maxpar^®^ system (Standard BioTools), which involves the site-specific conjugation of metal-chelating polymers to the antibodies via maleimide coupling. In order to achieve sensitivities that are comparable to conventional fluorescent flow cytometry, about 100 atoms of a given metal isotope should be bound to each antibody (Ornatsky et al. 2010, Tanner et al. 2013). This high metal loading facilitates sensitive detection of immune cell markers for single-cell immunophenotyping, which remains the most widespread application of CyTOF (Iyer et al. 2025, Sahaf et al. 2020).

In contrast, our approach employed random conjugation of the bifunctional chelator p-SCN-Bn-DOTA to the anti-CD30 antibody via lysine residues, resulting in a chelator-to-antibody ratio of approximately six. This strategy mirrors the conjugation methods commonly used in the development of radioimmunoconjugates for TRT, where DOTA-based chelating agents remain the most widely applied chelators due to their capacity to form stable complexes with radiolanthanides such as ^177^Lu (Baranyai et al. 2020, Stasiuk and Long 2013, Price and Orvig 2014). Typically, radioimmunoconjugates for TRT are designed with a limited number of chelators per antibody to preserve immunoreactivity and in vivo pharmacokinetics while ensuring sufficient specific activity (Delage et al. 2021, Knogler et al. 2006).

Despite the lower metal load compared to conventional CyTOF tagging systems, we successfully detected the uptake of the stable surrogate [^175^Lu]Lu-DOTA-cAC10 at the single-cell level across all the three tested cell lines. Notably, uptake was measurable even in Jurkat cells, which exhibited the lowest CD30 expression (Table 1; Fig. 3B). Furthermore, the differences observed in cellular uptake among the cell lines under study, as determined by mass cytometry, strongly correlate with the CD30 expression measured by flow cytometry and are in good concordance with the uptake data determined by γ-counting (Table 1). These results demonstrate that the cellular uptake of radioimmunoconjugates, despite incorporating a limited number of metal chelators, can be effectively evaluated at the single-cell level using mass cytometry strategies. However, the sensitivity of the method may limit the detection of uptake in off-target tissues or in cells with lower target expression. Nevertheless, as most radiopharmaceuticals target membrane proteins highly expressed on tumor cells, mass cytometry remains a valuable tool for their characterization.

Several studies have previously explored the analysis of radiopharmaceuticals at single-cell resolution. The use of conventional fluorescence microscopy has been reported, but it requires chemical modification of radiopharmaceuticals with fluorophores to visualize their cellular distribution and only a limited number of cells can be analyzed at the same time (Carlucci et al. 2015). Radioluminescence microscopy enables detection without fluorescent labeling, but it is not high-throughput and lacks multiplexing capabilities for simultaneous marker analysis (Pratx et al. 2012, Sengupta and Pratx 2016). Some studies have explored droplet encapsulation approaches (droplet radiofluidics) to improve multiplexing, but the method is technically challenging, thus applications remain limited (Türkcan et al. 2015). More recently, fluorescence activated cell sorting (FACS) has been used in combination with γ-counting (“RadioFlow”) to quantify [^18^F]FDG uptake in different cell populations (Vraka et al. 2025). In this study, mice bearing lung tumors were injected with [^18^F]FDG, and different cell populations, including cancer cells, macrophages, CD8^+^ and CD4^+^ T cells, were isolated by cell sorting. The radioactivity associated with each population was subsequently measured by γ-counting and normalized to the number of cells. While this approach provides improved resolution of radiotracer uptake across defined cell populations compared with conventional γ-counting of whole tumors or organs, the readout remains a bulk measurement, reflecting the average uptake of each population rather than uptake at the single-cell level. Consequently, intercellular heterogeneity within each population cannot be resolved. Furthermore, this method requires prior selection and sorting of predefined cell populations, which introduces additional handling steps, limits multiplexing capacity, and may cause bias, particularly for the detection of rare cell subpopulations. In contrast, mass cytometry allows multiplexing and single-cell resolution across different cell populations, providing individual cell uptake measurements and allowing the characterization of intra-population variability. A similar single-cell mass cytometry approach has been applied to investigate the interactions between immune cells and lanthanide-doped upconversion nanoparticles (UCNPs) with potential therapeutic applications (Gerelkhuu et al. 2023). In this study, mass cytometry enabled single-cell analysis of UCNPs interaction and toxicity in twelve immune cell types from human peripheral blood mononuclear cells (hPBMCs), revealing substantial heterogeneity in uptake both across and within cell populations, including the identification of immune sub-cell types with differential affinity for the nanoparticles. These findings highlight the relevance of single-cell methods to characterize heterogeneous interactions between pharmaceutical systems and complex biological environments. In this context, our study represents, to our knowledge, the first application of mass cytometry to evaluate radiopharmaceutical uptake at single-cell resolution. This well-established, high-throughput, and multiplexed analytical tool has the potential to significantly improve preclinical radiopharmaceutical characterization.

## Conclusion

We demonstrated that mass cytometry is a powerful analytical method for evaluating the single-cell uptake of radiopharmaceuticals using stable surrogates. The approach established here enabled the assessment of the non-radioactive analog [^175^Lu]Lu-DOTA-cAC10 uptake at single-cell resolution, revealing receptor-dependent accumulation and cellular heterogeneity, which is not detectable with conventional bulk analysis methods such as γ-counting. The ability to evaluate radiopharmaceutical distribution at the single-cell level holds the potential to improve preclinical radiopharmaceutical characterization and dosimetry assessment, allowing a more accurate evaluation of potential radiopharmaceutical accumulation in healthy organs.

## Supplementary Information

Below is the link to the electronic supplementary material.

Supplementary Material 1

## Data Availability

The datasets used and/or analyzed during the current study are available from the corresponding author on reasonable request.

## Abbreviations

DOTA: 1,4,7,10-Tetraazacyclododecane-1,4,7,10-tetraacetic acid
EDTA: Ethylenediaminetetraacetic acid
HPLC: High performance liquid chromatography
ICP-MS: Inductively coupled plasma mass spectrometry
LC-MS: Liquid chromatography mass spectrometry
SEC: Size exclusion chromatography
PET: Positron emission tomography
SPECT: Single photon emission computed tomography
TOF: Time of flight
CyTOF: Cytometry by time-of-flight
TRT: Targeted radionuclide therapy

## References

[CR18] Atkuri KR, Stevens JC, Neubert H. Mass cytometry: a highly multiplexed single-cell technology for advancing drug development. Drug Metab Dispos. 2015;43(2):227–33. 25349123 10.1124/dmd.114.060798

[CR30] Baranyai Z, Tircsó G, Rösch F. The use of the macrocyclic chelator DOTA in radiochemical separations. Eur J Inorg Chem. 2020;2020(1):36–56.

[CR8] Bejarano L, Jordāo MJC, Joyce JA. Therapeutic targeting of the tumor microenvironment. Cancer Discov. 2021;11(4):933–59. 33811125 10.1158/2159-8290.CD-20-1808

[CR16] Bendall SC, Nolan GP, Roederer M, Chattopadhyay PK. A deep profiler’s guide to cytometry. Trends Immunol. 2012;33(7):323–32. 22476049 10.1016/j.it.2012.02.010 PMC3383392

[CR33] Carlucci G, Carney B, Brand C, Kossatz S, Irwin CP, Carlin SD, et al. Dual-modality optical/PET imaging of PARP1 in glioblastoma. Mol Imaging Biol. 2015;17(6):848–55. 25895168 10.1007/s11307-015-0858-0 PMC4609241

[CR21] Chuah S, Chew V. High-dimensional immune-profiling in cancer: implications for immunotherapy. J Immunother Cancer. 2020;8(1):e000363. 32034066 10.1136/jitc-2019-000363 PMC7057482

[CR27] Cooper MS, Sabbah E, Mather SJ. Conjugation of chelating agents to proteins and radiolabeling with trivalent metallic isotopes. Nat Protoc. 2006;1(1):314–7. 17406251 10.1038/nprot.2006.49

[CR14] Corte RodríguezM, Álvarez-Fernández García R, Blanco E, Bettmer J, Montes-Bayón M. Quantitative evaluation of cisplatin uptake in sensitive and resistant individual cells by single-cell ICP-MS (SC-ICP-MS). Anal Chem. 2017;89(21):11491–7. 29023104 10.1021/acs.analchem.7b02746

[CR13] Corte-Rodríguez M, Álvarez-Fernández R, García-Cancela P, Montes-Bayón M, Bettmer J. Single cell ICP-MS using on line sample introduction systems: current developments and remaining challenges. TrAC Trends Anal Chem. 2020;132:116042.

[CR25] Delage JA, Faivre-Chauvet A, Barbet J, Fierle JK, Schaefer N, Coukos G, et al. Impact of DOTA conjugation on pharmacokinetics and immunoreactivity of [177Lu]Lu-1C1m-Fc, an Anti TEM-1 fusion protein antibody in a TEM-1 positive tumor mouse model. Pharmaceutics. 2021;13(1):96. 33451158 10.3390/pharmaceutics13010096 PMC7828678

[CR38] Gerelkhuu Z, Perumalsamy H, Maddahfar M, Jin D, Songe J, Yoon TH. A single-cell based mass cytometry study on heterogeneous interactions between upconversion nanoparticles and human immune cells. Environ Sci: Nano. 2023;10:824–33.

[CR28] Han G, Spitzer MH, Bendall SC, Fantl WJ, Nolan GP. Metal-isotope-tagged monoclonal antibodies for high-dimensional mass cytometry. Nat Protoc. 2018;13(10):2121–48. 30258176 10.1038/s41596-018-0016-7 PMC7075473

[CR4] Hennrich U, Kopka K. Lutathera®. The first FDA- and EMA-approved radiopharmaceutical for peptide receptor radionuclide therapy. Pharmaceuticals. 2019;12(3):114. 31362406 10.3390/ph12030114 PMC6789871

[CR20] Iyer A, Hamers AAJ, Pillai AB. CyTOF® for the masses. Front Immunol. 2022;14:13. 10.3389/fimmu.2022.815828 PMC9047695 35493491

[CR9] Klika KD, Han J, Busse MS, Soloshonok VA, Javahershenas R, Vanhaecke F, et al. Inductively Coupled Plasma-Mass Spectrometry (ICP-MS): An Emerging Tool in Radiopharmaceutical Science. J Am Chem Soc. 2024;146(45):30717–27. 39478417 10.1021/jacs.4c12254 PMC11565647

[CR26] Knogler K, Grünberg J, Novak-Hofer I, Zimmermann K, Schubiger PA. Evaluation of 177Lu-DOTA-labeled aglycosylated monoclonal anti-L1-CAM antibody chCE7: influence of the number of chelators on the in vitro and in vivo properties. Nucl Med Biol. 2006;33(7):883–9. 17045168 10.1016/j.nucmedbio.2006.08.001

[CR7] Lawhn-Heath C, Hope TA, Martinez J, Fung EK, Shin J, Seo Y, et al. Dosimetry in radionuclide therapy: the clinical role of measuring radiation dose. Lancet Oncol. 2022;23(2):e75–87. 35114134 10.1016/S1470-2045(21)00657-4

[CR3] Lepareur N, Ramée B, Mougin-Degraef M, Bourgeois M. Clinical advances and perspectives in targeted radionuclide therapy. Pharmaceutics. 2023;15(6):1733. 37376181 10.3390/pharmaceutics15061733 PMC10303056

[CR10] Liu Z, Li X, Xiao G, Chen B, He M, Hu B. Application of inductively coupled plasma mass spectrometry in the quantitative analysis of biomolecules with exogenous tags: a review. TrAC Trends Anal Chem. 2017;93:78–101.

[CR23] Maby P, Corneau A, Galon J. Phenotyping of tumor infiltrating immune cells using mass-cytometry (CyTOF). Methods Enzymol. 2020;632:339–68. 32000904 10.1016/bs.mie.2019.07.025

[CR19] Majonis D, Herrera I, Ornatsky O, Schulze M, Lou X, Soleimani M, et al. ynthesis of a functional metal-chelating polymer and steps toward quantitative mass cytometry bioassays. Anal Chem. 2010;82(21):8961–9. 20939532 10.1021/ac101901x PMC3056155

[CR6] Nelson BJB, Krol V, Bansal A, Andersson JD, Wuest F, Pandey MK. Aspects and prospects of preclinical theranostic radiopharmaceutical development. Theranostics. 2024;14(17):6446–70. 39479448 10.7150/thno.100339 PMC11519794

[CR15] Ornatsky O, Bandura D, Baranov V, Nitz M, Winnik MA, Tanner S. Highly multiparametric analysis by mass cytometry. J Immunol Methods. 2010;361(1–2):1–20. 20655312 10.1016/j.jim.2010.07.002

[CR34] Pratx G, Chen K, Sun C, Martin L, Carpenter CM, Olcott PD et al. Radioluminescence microscopy: measuring the heterogeneous uptake of radiotracers in single living cells. PLoS ONE. 2012;7(10):e46285. 23056276 10.1371/journal.pone.0046285 PMC3463617

[CR32] Price EW, Orvig C. Matching chelators to radiometals for radiopharmaceuticals. Chem Soc Rev. 2014;43(1):260–90. 24173525 10.1039/c3cs60304k

[CR24] Rioja-Blanco E, Banz Y, Schlapbach C, Novak U, Chiorazzo T, Bertschi NL, et al. 161Tb radioimmunotherapy as a treatment for CD30-Positive Lymphomas. J Nucl Med. 2025;66(6):909–15. 40456588 10.2967/jnumed.124.268805 PMC12175992

[CR29] Sahaf B, Rahman A, Maecker HT, Bendall SC. High-parameter immune profiling with CyTOF. In: Thurin M, Cesano A, Marincola F, editors. Biomarkers for Immunotherapy of Cancer. Methods in Molecular Biology. New York, NY: Humana; 2020. 10.1007/978-1-4939-9773-2_16 PMC7232871 31502160

[CR5] Sallam M, Nguyen NT, Sainsbury F, Kimizuka N, Muyldermans S, Benešová-Schäfer M. PSMA-targeted radiotheranostics in modern nuclear medicine: then, now, and what of the future? Theranostics. 2024;14(8):3043–79. 38855174 10.7150/thno.92612 PMC11155394

[CR35] Sengupta D, Pratx G. Single-Cell Characterization of 18F-FLT Uptake with radioluminescence microscopy. J Nucl Med. 2016;57(7):1136–40. 27081170 10.2967/jnumed.115.167734 PMC5293188

[CR2] Sgouros G, Bodei L, McDevitt MR, Nedrow JR. Radiopharmaceutical therapy in cancer: clinical advances and challenges. Nat Rev Drug Discov. 2020;19(9):589–608. 32728208 10.1038/s41573-020-0073-9 PMC7390460

[CR31] Stasiuk GJ, Long NJ. The ubiquitous DOTA and its derivatives: the impact of 1,4,7,10-tetraazacyclododecane-1,4,7,10-tetraacetic acid on biomedical imaging. Chem Commun. 2013;49(27):2732. 10.1039/c3cc38507h 23392443

[CR17] Tanner SD, Baranov VI, Ornatsky OI, Bandura DR, George TC. An introduction to mass cytometry: fundamentals and applications. Cancer Immunol Immunother. 2013;62(5):955–65. 23564178 10.1007/s00262-013-1416-8 PMC11029414

[CR36] Türkcan S, Nguyen J, Vilalta M, Shen B, Chin FT, Pratx G, et al. Single-cell analysis of [18F]fluorodeoxyglucose uptake by droplet radiofluidics. Anal Chem. 2015;87(13):6667–73. 26035453 10.1021/acs.analchem.5b00792 PMC4669076

[CR1] Varghese TP, John A, Mathew J. Revolutionizing cancer treatment: the role of radiopharmaceuticals in modern cancer therapy. Precis Radiat Oncol. 2024;8(3):145–52. 40336977 10.1002/pro6.1239 PMC11935213

[CR37] Vraka C, Homolya M, Özer Ö, Spittler A, Machtinger M, Moll HP, et al. Radioflow cytometry reveals That [^18^F]FDG uptake in K-RAS lung cancer is driven by immune cells: an analysis on a single-cell level. J Nucl Med. 2025;66(2):215–22. 39819684 10.2967/jnumed.124.268799 PMC11800735

[CR12] Wallimann RH, Hensinger H, Müller C, Schibli R, Kneuer R, Schindler P. Liquid chromatography ICP-MS to assess the stability of 175Lu- and natGa-based tumor-targeting agents towards the development of 177Lu- and 68Ga-labeled radiopharmaceuticals. Pharmaceutics. 2024;16(3):299. 38543193 10.3390/pharmaceutics16030299 PMC10974936

[CR11] Wallimann RH, Schindler P, Hensinger H, Tschan VJ, Busslinger SD, Kneuer R, et al. Inductively coupled plasma mass spectrometryA valid method for the characterization of metal conjugates in view of the development of radiopharmaceuticals. Mol Pharm. 2023;20(4):2150–8. 36826437 10.1021/acs.molpharmaceut.2c01092

[CR22] Zhang T, Warden AR, Li Y, Ding X. Progress and applications of mass cytometry in sketching immune landscapes. Clin Transl Med. 2020. 10.1002/ctm2.206. 33135337 10.1002/ctm2.206 PMC7556381

