# Supplementary material for: The use of mass cytometry (CyTOF) to evaluate the cellular uptake of stable radiopharmaceutical surrogates in single cells: a proof-of-concept study
Source: EJNMMI Radiopharm Chem. 2026 Apr 1;11:22. doi: 10.1186/s41181-026-00442-2 (PMC13043783; doi:10.1186/s41181-026-00442-2)
Supplement: Supplementary file 1 — Supplementary Material 1 [file 41181_2026_442_MOESM1_ESM.docx]

**Supporting information for:**

**The use of mass cytometry (CyTOF) to evaluate the cellular uptake of stable radiopharmaceutical surrogates in single cells: a proof-of-concept study**

Miguel Gómez-Sánchez^1,2^, Elisa Blanco-González^1,2^, María Montes- Bayón^1,2^, Martin Behe^3^, Roger Schibli^3,4^, Elisa Rioja-Blanco^3,4*^

1. Department of Physical and Analytical Chemistry, Faculty of Chemistry, University of Oviedo, Julián Clavería 8, Oviedo 33006, Spain
2. Instituto de Investigación Sanitaria del Principado de Asturias (ISPA), Av. Hospital Universitario s/n, Oviedo 33011, Spain
3. Center for Radiopharmaceutical Sciences, PSI Center for Life Sciences, Villigen-PSI, Switzerland.
4. Department of Chemistry and Applied Biosciences, Institute of Pharmaceutical Sciences, ETH Zurich, Zurich, Switzerland.

* Corresponding author:

Elisa Rioja-Blanco: Tel: +41563102178; E-Mail: elisa.rioja-blanco@psi.ch

**
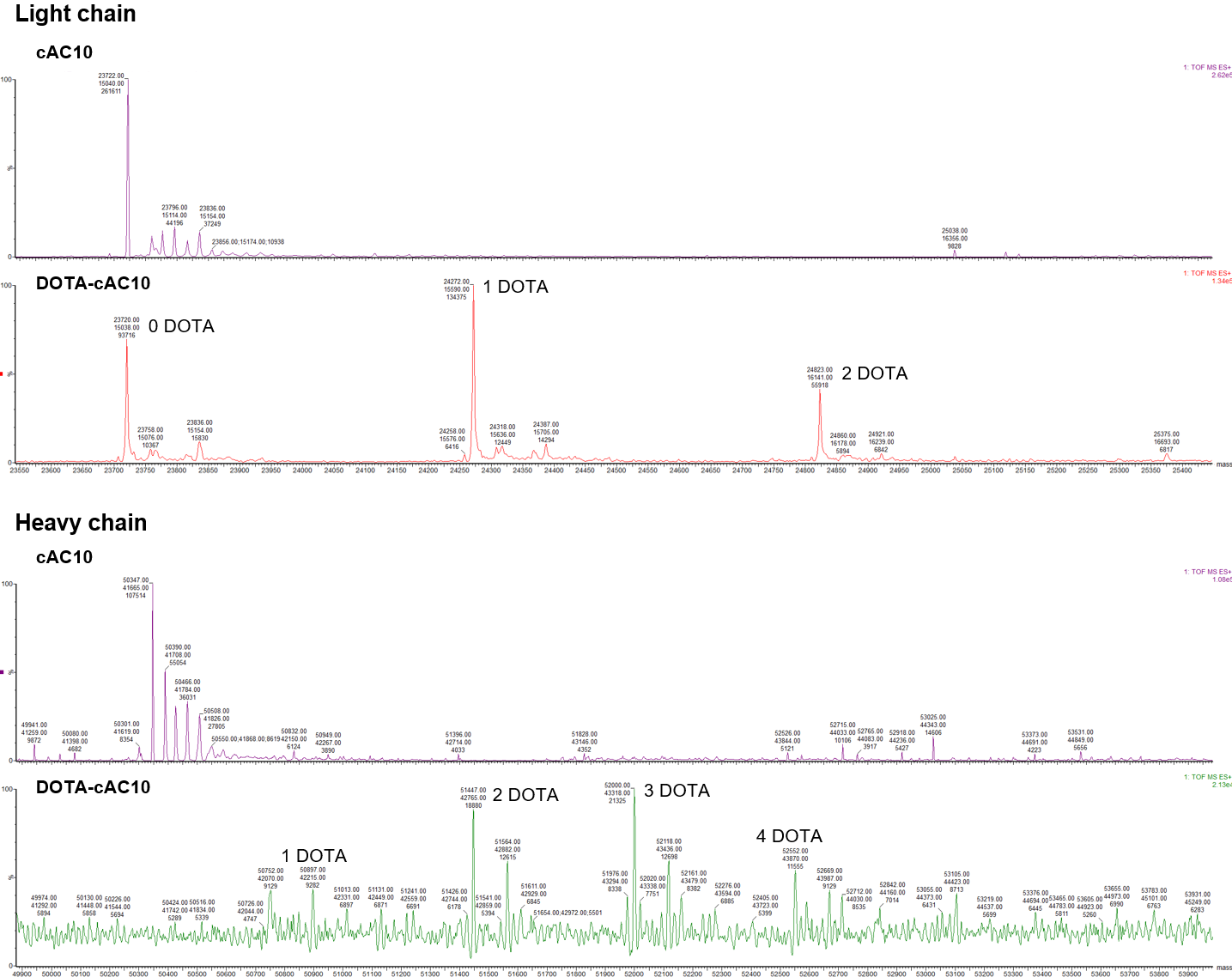
**

**Figure S1.** MaxEnt1 deconvoluted mass spectra of the reduced cAC10 antibody and the DOTA-cAC10 immunoconjugate.


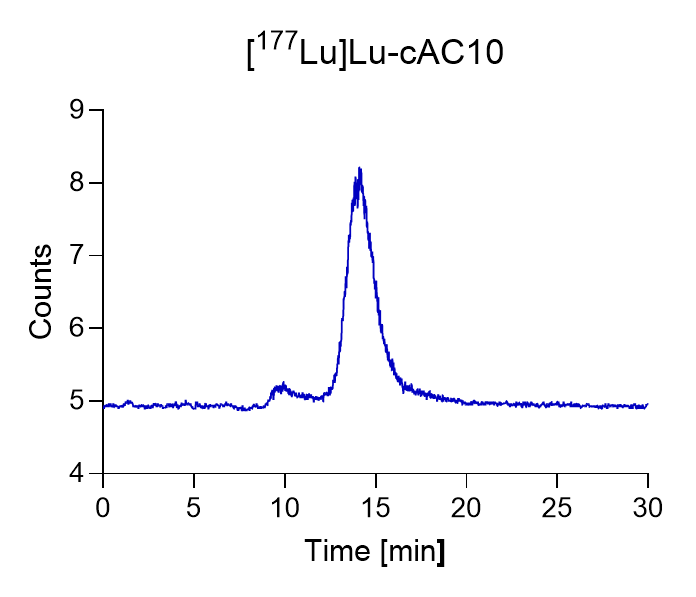


**Figure S2.** Representative SEC chromatogram with on-line radiodetection of the immune conjugate DOTA-cAC10 labeled with ^177^Lu after PD10 purification. The main peak at ~14 min corresponded to [^177^Lu]Lu-DOTA-cAC10. The peak at ~10 min was assigned to radiolabeled antibody aggregates.


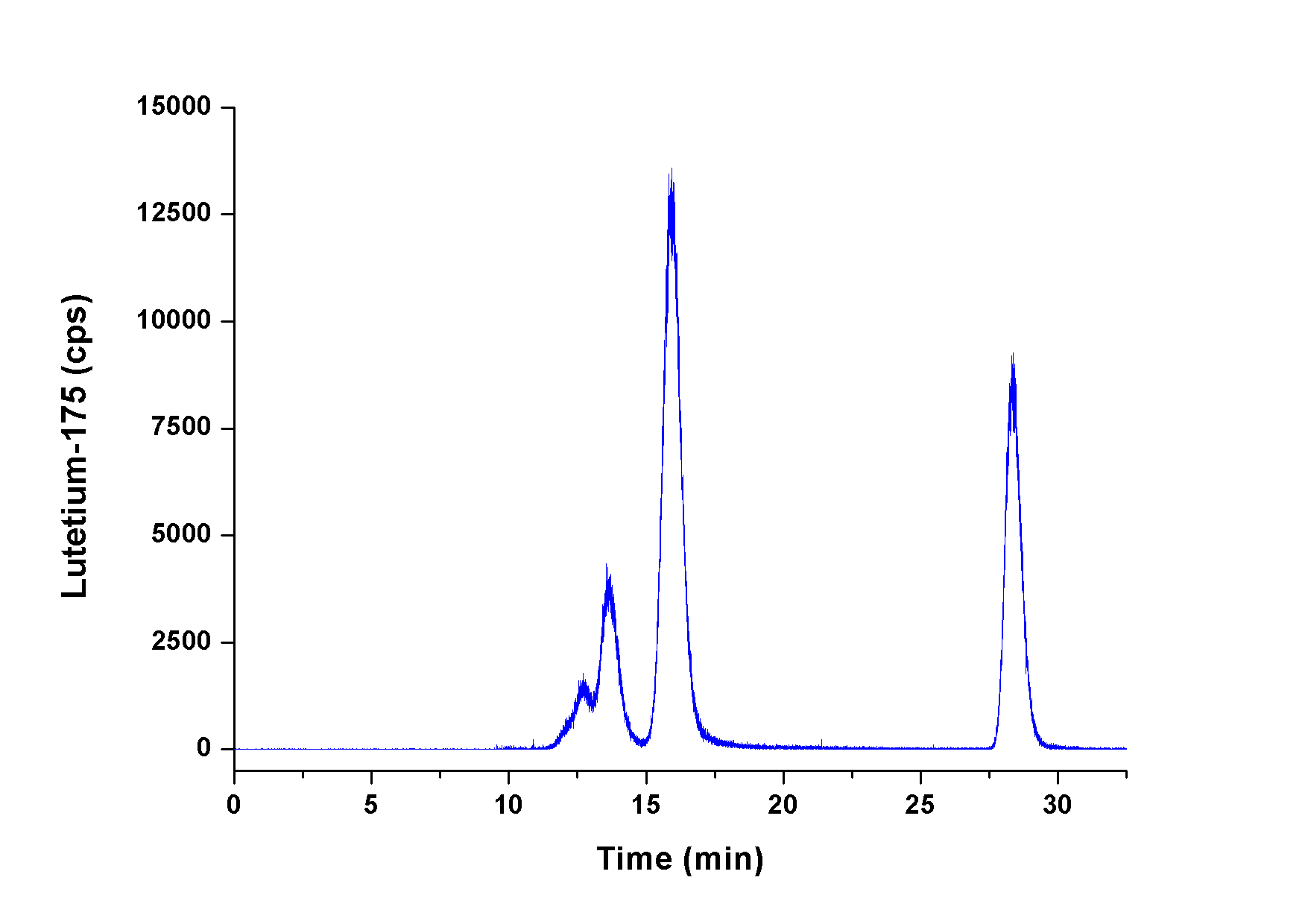


**Figure S3.** Representative SEC-ICP-MS chromatogram monitoring ^175^Lu^+^ of the immune conjugate DOTA-cAC10 labeled with ^175^Lu. The peak at ~16 min corresponded to [^175^Lu]Lu-DOTA-cAC10. The peaks at 12-14 min were assigned to metal-labeled antibody aggregates. The peak at ~28 min corresponded to the complex of ^175^Lu with EDTA.


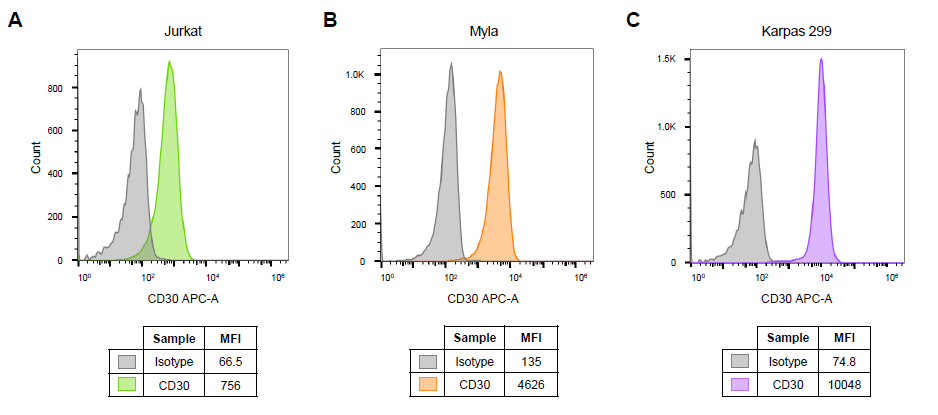


**Figure S4.** CD30 membrane expression in the three different T-cell lymphoma cell lines by flow cytometry using the anti-CD30- allophycocyanin (APC) antibody for staining. Histograms of the CD30-stained cell population in Jurkat (in green) (A), Myla (in orange) (B), and Karpas 299 (in violet) (C) cells lines in comparison with the isotype controls (in grey).

**
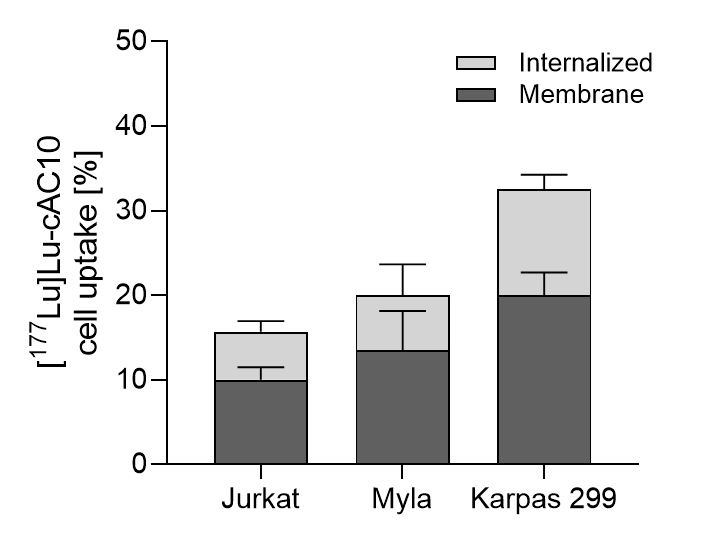
**

**Figure S5.** Percentage of [^177^Lu]Lu-DOTA-cAC10 cellular uptake in the membrane and internalized fractions for the three tested cell lines measured by γ-counting (mean ± SD, n=3).


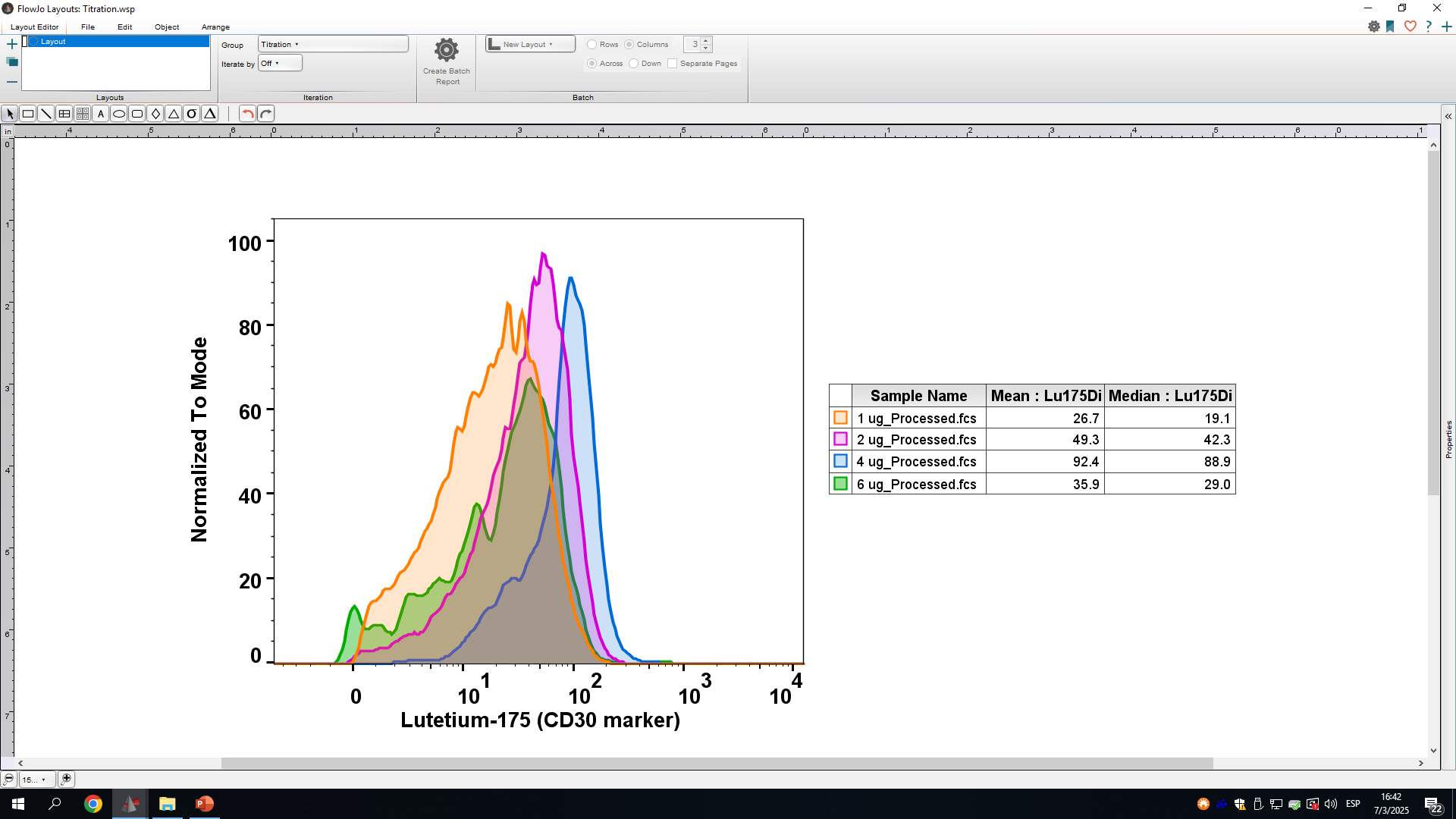


**Figure S6.** Overlay histograms of the ^175^Lu signal (normalized to the mode, most frequent value) obtained by CyTOF on Karpas 299 cells treated with [^175^Lu]Lu-DOTA-cAC10 at different concentrations: 1µg/mL (in orange), 2 µg/mL (in pink), 4 µg/mL (in blue) and 6 µg/mL (in green).
